# Colorectal Cancer in Brunei Darussalam: An Overview and Rationale for National Screening Programme

**DOI:** 10.31557/APJCP.2019.20.12.3571

**Published:** 2019

**Authors:** Mei Ann Lim, Vui Heng Chong, Sok King Ong, Ya Chee Lim

**Affiliations:** 1 *PAPRBS Institute of Health Sciences, Universiti Brunei Darussalam, *; 2 *Ministry of Health, Bandar Seri Begawan, Brunei Darussalam. *

**Keywords:** Colorectal cancer, national screening, risk factors, Brunei Darussalam

## Abstract

Colorectal cancer (CRC) is the third most common cancer worldwide after lung and breast cancers, and ranks second in terms of cancer mortality globally. Brunei Darussalam reports high incidence of CRC in the Southeast Asian region and has no formal national screening programme for CRC. Screening for CRC in Brunei Darussalam is offered in an opportunistic fashion for individuals with average or above average risks for CRC, that is, the individual has a positive family history of CRC or neoplasms and is more than 50 years old. Opportunistic screening is widely practiced but this is not standardised. The Ministry of Health in Brunei Darussalam is currently in the process of implementing a CRC screening programme as part of a larger national health screening based on the increasing incidence of non-communicable diseases (NCDs). This review article assesses the situation of CRC in Brunei Darussalam from the 1980s to present day, including incidence of CRC in different age groups, ethnicities and genders; relevant non-modifiable and modifiable risk factors of CRC in Brunei Darussalam setting; and common CRC screening techniques used in Brunei Darussalam as well as other Asia-Pacific countries. The review also discusses the merits of a national CRC screening programme. With the increasing incidence of CRC worldwide and in Brunei Darussalam, national screening for CRC in Brunei Darussalam is an important strategy to lower morbidity and mortality rates. A review of the progress and outcome of the national screening programme will be available a few years after rollout.

## Introduction

Colorectal cancer (CRC) is the third most common cancer worldwide after lung and breast cancers, and ranks second in terms of cancer mortality (Bray et al., 2018). According to GLOBOCAN 2018 estimates, worldwide CRC is the third most common cancer in men after lung and prostate, with about 1.03 million new cases reported in 2017 (10.9% of total new cancer incidence for men), while CRC is the second most common in women after breast cancer, with reported 0.82 million new cases in 2017 (9.5% of total new cancer incidence for women). There is generally some variation in the incidence rates across the world, but the patterns are very similar in men and women. The incidence rates correlate with the level of development of the nations; hence, CRC is a condition associated with affluence. The incidence rates have been steadily increasing in the developing nations, with some reported rates approaching those of developed nations. Brunei Darussalam, one of the member states of the Association of Southeast Asian Nations (ASEAN), is also seeing steady increase in the incidence of CRC, with the latest rate reported to be 42.6 per 100,000 population for males and 32.9 per 100,000 population for females, both high figures in the Southeast Asian region (Ong et al., 2018). The lifetime risks of developing CRC for both males and females in Brunei Darussalam are higher than those for both genders in neighbouring Malaysia and Singapore (Ong et al., 2018). Worldwide CRC accounted for about 881,000 deaths (9.2% of total cancer mortality) in 2018 (Bray et al., 2018). 

In the world’s current largest and longest ever clinical trial for the prevention of CRC, the UK Flexible Sigmoidoscopy Screening Trial (UKFSST), a single screening flexible sigmoidoscopy is reported to significantly reduced CRC incidence and mortality rates over a period of at least 17 years (Atkin et al., 2017). This flexible sigmoidoscopy screening programme was rolled out across England for those aged ≥ 55 years and is a part of the NHS Bowel Cancer Screening Programme (BCSP), which also includes biennial guaiac faecal occult blood tests (gFOBT) for those aged between 60 and 74 years (Atkin et al., 2017; Mayor, 2011). The NHS BCSP is consistent in most parts with the US Preventive Services Task Force (USPSTF) recommendation of screening for CRC using FOBT, sigmoidoscopy or colonoscopy, beginning at age 50 years and continuing until the age 75 years. According to USPSTF, CRC screening for those aged 76 years old and above is appropriate if these adults are healthy enough to undergo treatment should CRC be found; if they do not have comorbid conditions that would already limit their life expectancy; and if they have never been screened for CRC (Ngo-Metzger and Rajupet, 2017). About half of the countries belonging to the European Union have CRC screening programmes in place (Altobelli et al., 2014). 

In Brunei Darussalam, FOBT using faecal immunochemical test (FIT) and CRC screening by colonoscopy are practised. However, there is currently no formal national CRC screening programme, and screening is offered in an opportunistic fashion for individuals with average or above average risks for CRC, that is, the individual has a positive family history of CRC or neoplasms and is more than 50 years old. This review article assesses the situation of CRC in Brunei Darussalam and discusses the merits of a CRC screening programme. 

*Natural History, Symptoms and Risk Factors of CRC*

*Natural History of CRC*

All CRCs arise from a precursor lesion, in the form of an abnormal outgrowth from the colorectal lining, called the adenomatous polyp or adenoma. Adenomas exhibit different growth patterns, and as such, can be classified under different names: tubular, villous, tubulovillous, sessile serrated and traditional serrated. Polyps also exhibit different behaviours – they may be benign neoplastic (hyperplasia), pre-cancerous/pre-malignant *in situ* neoplasm (dysplasia), or cancerous/malignant (colorectal adenocarcinoma). Polyps can be elevated, flat or depressed. Flat and depressed polyps are typically lateral spreading. 

Approximately 95% of CRCs are adenocarcinomas (cancer of glandular epithelial cells), while the other types include mucinous carcinomas (cancer of mucin-producing epithelial cells) and adeno-squamous carcinomas (cancer of both squamous and gland-like cells) (WCRF/AICR, 2018). This pattern is also observed in Brunei Darussalam (Chong et al., 2009). The most common CRC in Brunei Darussalam is adenocarcinoma (97.2%) followed by neuroendocrine tumour (1.4%), and lymphoma (0.9%), with rectum accounting for a third of CRC site (Koh et al., 2015).

*Symptoms of CRC*

In the early stages of CRC, patients are asymptomatic or have mild, non-specific gastrointestinal symptoms. Presence of symptoms indicate that the disease has been present for several years. Common CRC symptoms include altered bowel habit, bleeding per rectum, non-specific abdominal pain, symptoms of anaemia, weight loss and loss of appetite. In Brunei Darussalam, the most common indication for colonoscopy referral is bleeding per rectum (Chong et al., 2012). However, symptoms such as abdominal pain, which are typically non-specific, and bleeding per rectum are common in the community. In most cases, these symptoms are self-limiting, often due to non-malignant disorders, and hence, not necessarily requiring any endoscopic investigations (Adelstein et al., 2011; Chong, 2008); nonetheless, blood in stool still warrant investigation for ‘any disease worth identifying’ and not just CRC alone (Adelstein et al., 2011; Astin et al., 2011).

Left-sided colon or rectal cancer tend to present with altered bowel habit (typically loose stool) and bleeding per rectum, with or without pain. Right-sided lesions tend to present with anaemia, with or without pain. Symptoms such as weight loss, anorexia, abdominal distention, nausea and vomiting are symptoms of advanced diseases. However, even in advanced disease, a patient may not report or perceive any symptoms as symptoms tend to develop over a prolonged period. 

*Risk Factors of CRC*

Risk factors for CRC consist of non-modifiable and modifiable factors. Examples of non-modifiable risk factors are age, genetic predispositions (family history of CRC or related cancer syndrome), and specific large bowel diseases such as inflammatory bowel diseases (IBDs). Non-modifiable risk factors are factors that cannot be altered, with the exception for IBDs where control of this disease can be greatly influenced. The modifiable risk factors are largely related to individual habits and lifestyle that are influenced by cultural and social factors. Important components of modifiable risk factors are poor dietary habit, low physical activity, and their subsequent complications. In general, it is recommended to maintain a healthy weight, to be physically active, and to eat a healthy diet (WCRF/AICR, 2018).

*Non-Modifiable Risk Factors*

The main non-modifiable risk factors for CRC are increasing age, genetic predispositions (positive family history of CRC and other related cancers cluster), previous history of polyps, and IBDs (Crohn’s disease and Ulcerative Colitis) (Cunningham et al., 2010; WCRF/AICR, 2018). In Brunei’s setting, age and positive family history of CRC or polyps are taken into account for indication. The mean age of CRC diagnosis in Brunei Darussalam was 59.3 ± 14.6 years old (Chong et al., 2015b), and the age group found to have the highest incidence of CRC for both men and women was 55-59 years (Ong et al., 2018). 

Two well-established forms of CRC due to genetic risk factors are Familial Adenomatous Polyposis (FAP) and Hereditary Non-Polyposis CRC (HNPCC) (Cunningham et al., 2010; WCRF/AICR, 2018). Germinal and somatic mutations in the adenomatous polyposis coli (APC) gene, a key tumour suppressor, have been identified to be the root cause for FAP, a condition that predisposes a person to develop numerous, typically hundreds of adenomatous polyps in the bowels, specifically the large bowel (Aoki and Taketo, 2007). Germline mutations in DNA mismatch repair (MMR) genes, mainly MLH1, MSH2 and MSH6, are responsible for HNPCC, also known as Lynch Syndrome, a condition that increases the risk for CRC (Ramsoekh et al., 2009). Based on Brunei’s national registries on endoscopy, pathology and cancer, there are currently less than seven families with documented familial cancer syndrome in Brunei Darussalam; one family with attenuated FAP, two patients with FAP but without family history of CRC (likely index cases), and two HNPCC syndrome. In Brunei Darussalam, genetic testing for FAP and HNPCC is selectively only for known family history of CRC syndrome and is referred to overseas centres for testing.

The incidence of IBDs remains low but there is an increasing trend, and this trend is seen worldwide (Ng et al., 2018) and in Brunei Darussalam (Ong et al., 2018). Generally, manifestations of IBDs in the Asian setting are milder than those reported from the West (Wang et al., 2007). Based on Brunei’s national IBD registry, there has not been any IBD patients who, upon follow up, have been diagnosed with CRC (Ong et al., 2018). 

**Table 1:** Incidence and Lifetime Risk of Bruneians (citizens and permanent residents) for Years 2011 to 2015 in Brunei Darussalam by Gender and Cancer Types (adapted from S K Ong et al., 2018). CR, crude rate of cancer incidence; ASR, age-standardised rate of cancer incidence

Gender	Cancer Type	CR per 100,000	ASR per 100,000	Lifetime Risk	
				%	1 in *x* persons
Overall	All cancers	172.9	241.0	28.0	4
Male	All cancers	144.11	222.6	26.5	4
	Colorectal	28.8	42.6	5.0	20
	Lung	19.2	32.2	4.5	22
	Prostate	12.7	24.2	3.7	27
Female	All cancers	201.7	258.5	29.2	3
	Breast	48.3	58.9	6.3	16
	Colorectal	24.1	32.9	4.5	22
	Cervical	18.5	20.3	1.8	56

**Table 2:** Incidence of CRC and other Cancer Types (trachea, bronchus and lung; breast; liver and intrahepatic bile duct; female cervical; male prostate) among Bruneians (citizens and permanent residents) for the Years 2005 to 2018 in Brunei Darussalam. *Percentages of cancer incidence due to CRC are given in parenthesis. (Source: Brunei Darussalam Cancer Registry).

Year	Incidence of Cancer Among Citizens and Permanent Residents of Brunei					
	Colorectal*	Trachea, Bronchus & Lung	Breast (Male & Female)	Liver & Intrahepatic Bile Duct	Cervical (Female) / Prostate (Male)	All Cancer Types
2005	36 (10.8%)	64	37	7	22 / 12	333
2006	42 (11.9%)	47	45	17	20 / 8	354
2007	35 (10.9%)	39	47	16	24 / 14	321
2008	39 (10.0%)	39	50	14	33 / 22	389
2009	41 (9.5%)	48	64	23	34 / 31	432
2010	69 (12.3%)	71	87	18	24 / 29	560
2011	79 (15.6%)	33	67	15	27 / 18	507
2012	79 (14.8%)	61	74	22	28 / 19	533
2013	88 (15.6%)	70	77	31	31 / 18	563
2014	78 (14.1%)	65	81	29	29 / 21	554
2015	106 (16.6%)	71	91	30	35 / 26	640
2016	105 (15.8%)	66	102	20	30 / 21	664
2017	101 (16.1%)	58	82	23	32 / 30	626
2018	102 (15.8%)	49	102	24	30 / 18	644

**Table 3:** Number of Deaths Due to All Cancer Types (including CRC) and other NCDs (heart diseases; diabetes mellitus; cerebrovascular diseases) for the Years 2005 to 2017 in Brunei Darussalam. *Percentages of total death in Brunei due to cancer is given in parenthesis. (Sources: Brunei Darussalam Cancer Registry; Health Information Booklets 2009 to 2017, Brunei Darussalam Ministry of Health.)

Year	Number of Deaths (Based on ICD-10)			
	All Cancer Types*	Heart Diseases	Diabetes Mellitus	Cerebrovascular Diseases
	(C00-C97)	(I00-I09; I20-I52)	(E10-E14)	(I60-I69)
2005	145 (13.5%)	176	118	71
2006	49 (4.5%)	188	116	102
2007	57 (4.9%)	177	140	87
2008	129 (11.8%)	211	97	93
2009	215 (18.4%)	185	100	97
2010	257 (21.3%)	186	100	99
2011	251 (20.3%)	183	116	86
2012	276 (22.7%)	152	123	70
2013	311 (22.2%)	183	131	82
2014	310 (21.1%)	228	141	103
2015	346 (22.4%)	193	147	102
2016	375 (23.0%)	211	149	123
2017	380 (22.4%)	250	171	143

**Table 4:** Number of Deaths Due to CRC and other Cancer Types (trachea, bronchus and lung; breast; liver and intrahepatic bile duct; female cervical; male prostate) for the Years 2005 to 2018 in Brunei Darussalam. *Percentages of cancer-related death in Brunei Darussalam due to CRC are given in parenthesis. (Source: Brunei Darussalam Cancer Registry.)

Year	Number of Deaths					
	Colorectal*	Trachea, Bronchus & Lung	Breast (Male & Female)	Liver & Intrahepatic Bile Duct	Cervical (Female) / Prostate (Male)	All Cancer Types
2005	14 (9.7%)	48	6	7	5 / 4	145
2006	4 (10.2%)	12	4	4	1 / 0	49
2007	6 (10.5%)	17	2	3	3 / 2	57
2008	17 (13.2%)	31	5	11	10 / 2	129
2009	25 (11.6%)	38	13	24	15 / 12	215
2010	35 (13.6%)	54	19	24	10 / 9	257
2011	38 (15.1%)	40	22	13	6 / 14	251
2012	43 (15.6%)	42	21	23	8 / 14	276
2013	39 (12.5%)	60	23	23	15 / 20	311
2014	40 (12.9%)	53	29	24	12 / 13	310
2015	47 (13.6%)	54	40	25	10 / 10	346
2016	53 (14.1%)	60	43	17	13 / 12	375
2017	70 (18.4%)	53	39	21	16 / 20	380
2018	41 (16.8%)	36	26	14	7 / 8	244

*Modifiable Risk Factors*

Modifiable risk factors of CRC are mainly associated with dietary patterns (red meat and processed food consumption, fibre consumption, and methods of food preparation), lifestyle (smoking, alcohol consumption, and sedentariness) and obesity (Cunningham et al., 2010; WCRF/AICR, 2018). Carcinogens is classified by the International Agency for Research on Cancer (IARC) into the following categories: group 1 – carcinogenic to humans; group 2A – probably carcinogenic to humans; group 2B – possibly carcinogenic to humans; group 3 – not classifiable as to its carcinogenicity to humans; and group 4 – probably not carcinogenic to humans (IARC, 2006). For example, tobacco smoking is a group 1 carcinogen in colorectal sites according to IARC (Cogliano et al., 2011). 

With regards to dietary patterns, the consumption of processed meat (meat preserved by smoking, curing, salting or adding chemical preservatives) and the intake of alcoholic beverages (intakes above 30 grams per day, equivalent to approximately two or more alcoholic drinks per day) as strong convincing risk factors of CRC (WCRF/AICR, 2018) is consistent with the findings reported by the IARC monograph series (Cogliano et al., 2011), which established that the consumption of processed meat and alcohol are group 1 carcinogens in CRC cases. The consumption of red meat (beef, pork, lamb and mutton) is a group 2 carcinogen (Cogliano et al., 2011; WCRF/AICR, 2018). There is limited suggestive evidence that low consumption of non-starchy vegetables and fruits (below 100 grams per day) might increase the risk of CRC (WCRF/AICR, 2018). In terms of protective dietary factors, there is strong probable evidence that foods containing wholegrains, dietary fibre, dairy products, and calcium supplements (above 200 milligrams per day) protect against CRC, while there is limited suggestive evidence that fish, vitamin D, and multivitamin supplements might decrease the risk of CRC (WCRF/AICR, 2018). Consuming food with vitamin C might decrease the risk of colon cancer (WCRF/AICR, 2018).

According to Heigi Library, there has been a general increasing trend in meat consumption in Brunei Darussalam from 1961 to 2013. Meat consumption is higher than fish consumption for almost all years from 1961 to 2013. Although fish consumption remains a common part of the diet in Brunei Darussalam, data from Food and Agriculture Organization Statistics (FAOSTAT) of the United Nations and Heigi Library suggest that there is a general decrease trend in fish consumption per capita in Brunei Darussalam between 1993 and 2013. Consumption of processed foods, associated with lower fibre intake and vitamins, has been high in Asia (Baker and Friel, 2014) and similarly in Brunei Darussalam, with more than 90% of the adult population not meeting the recommended daily consumption of fruits and vegetables (Ong et al., 2017). Studies among university students in Brunei Darussalam reported low daily consumption of fruits and vegetables (less than two servings daily), reflecting poor dietary habit (Sharif et al., 2016) despite good understanding regarding the importance of good nutrition (Tok et al., 2018). Eating choices are part of behavioural patterns with strong influence exerted from social factors (Kamarul et al., 2017). There are national programmes promoting healthy diet choices, including the implementation of healthy food guidelines in school canteens to combat childhood obesity. However, compliance by food vendors in schools has been challenging (Ahmad et al., 2013). Although alcohol consumption is a cause of CRC, this is generally not an issue in Brunei Darussalam due to strict restrictions on alcohol consumption in the country. Vitamin D deficiency is also generally not considered an issue, except in the older or younger population with limited sunlight exposure. 

The dietary pattern in Brunei Darussalam above is also reflected in the rate of obesity in Brunei Darussalam, which is among the highest in the Southeast Asian region (Ong et al., 2017). According to the Global School-based Student Health Survey (GSHS) for Brunei Darussalam in 2014, out of the 2599 students aged 13-17 years who participated in the survey, 35.2% were found to be overweight while 17.4% were obese (CDC and WHO, 2014). This would be a concern because greater body fatness (high body mass index and waist circumference or waist-hip ratio) is a convincing cause of CRC (IARC, 2014; WCRF/AICR, 2018). 

Physical activity of all types (occupational, household, transport and recreational) convincingly protects against colon cancer, but this conclusion is not extended to rectal cancer (IARC, 2014; WCRF/AICR, 2018). An additional source of concern is the level of physical activity in Brunei Darussalam. Among 353 primary school children, despite 93.8% of them being aware of the health benefits of physical activity, almost half (46.9%) did not meet the recommended amount of physical activity (Murang et al., 2017).

*CRC in Brunei Darussalam*

Based on two earlier studies using tissue-proven CRC made by the Brunei Darussalam state laboratory and a later study based on the Brunei Darussalam National Cancer Registry (which includes diagnosis made by other laboratories, typically oversea centres), cases of CRC in Brunei Darussalam is concluded to be increasing (Chong et al., 2009; Chong et al., 2015b; Ong et al., 2018). Over three time periods, the age-standardised rate (ASR) increased from 16.0 per 100,000 (1991-1998) to 19.6 per 100,000 (1999-2006) and to 24.3 per 100,000 (2007-2014) (Chong et al., 2015b). The latest study based on the established national cancer registry reported a higher ASR of 42.6 per 100,000 men and 32.9 per 100,000 women from 2011 to 2015, which translates to lifetime risks of 5.02% (for men) and of 4.47% (for women) in developing CRC (Table 1). From 2011 to 2018, CRC is the one of the top three frequent cancers alongside breast cancer and cancer of the trachea, bronchus and lungs among Bruneian citizens and permanent residents (Table 2). According to the national census, Brunei’s population was 421,300 people in 2017. Therefore, the national level of cancer burden due to CRC in terms of cancer incidence is high.

Among the ethnic groups (Malay, Chinese and indigenous), the Chinese showed the highest ASR (Chong et al., 2009). Young onset CRC (aged ≤ 45 years) was high (15.1%) compared to rates reported in the more developed nations (~5%) (Koh et al., 2015). The proportion of young onset CRC declined over the years, from 29.0% (1986-1990) to 13.2% (2011-2014) (Koh et al., 2015). The proportion of young onset CRC was highest among the indigenous (30.8%), followed by the expatriates (29.3%), Malays (14.3%), and lowest among the Chinese (10.8%) (Koh et al., 2015).

Increasing cases of CRC is also reflected by the increase in polyps detected by colonoscopy in clinical practice in Brunei Darussalam. Colonic polyps, the most common non-advanced polyps, and CRC were shown to have increased over two time periods, from 17.6% to 28.2% and from 4.0% to 4.7%, respectively (Chong et al., 2012; Wong et al., 2016). Colon tumours accounted for two-thirds of CRC, and there was no significant difference in tumour locations (colon or rectum) between non-young and young onset CRC cases (Koh et al., 2015). 

In terms of age at diagnosis among all CRC cases, the Chinese were older at diagnosis (mean age 62.6 years), compared to the Malays (mean age 58.4 years), and the indigenous (mean age 53.0 years) (Chong et al., 2009). These mean ages at time of CRC diagnosis can be contemplated in the context of a Bruneian’s life expectancy – in 2012, healthy life expectancy in both male and female Bruneians was 68 years old, which is nine years lower than overall life expectancy at birth, 77 years old. The difference of 9 years means an equivalent of nine years of full health lost through years lived with morbidity and disability. Among young onset CRC (aged ≤ 45 years), the mean age at diagnosis was 35.9 ± 6.2 years; oldest among the Chinese (38.6 ± 4.6), then Malays (35.6 ± 6.5), expatriates (34.9 ± 6.0), and lowest among the indigenous (33.5 ± 6.7), without any significant variation between the various population groups (p=0.056). A similar trend is observed for the Chinese (highest 65.9 ± 11.4) and Malay (second highest 62.8 ± 11.4) groups in the non-young onset CRC groups. While there was no significant difference in gender between young and non-young onset CRC cases, female young onset CRC was significantly younger than male young onset CRC (female mean age at diagnosis 34.7 years ± 6.7; male mean age at diagnosis 36.8 ± 5.7; p=0.029) (Koh et al., 2015). 

Cancer burden in Brunei Darussalam in terms of cancer incidence is high, and it is also high with respect to cancer mortality. From years 2005 to 2017, the four leading causes of death which consistently accounted for more than 50% of total deaths in Brunei Darussalam annually were comprised of non-communicable diseases (NCDs). Based on the 10th revision of the International Statistical Classification of Diseases and Related Health Problems (ICD-10), these NCDs are cancer (C00-C97), heart diseases (I00-I09, I20-I52), diabetes mellitus (E10-E14) and cerebrovascular diseases (160-I169). Since 2009, cancer was the nation’s top cause of deaths, with the percentages of total deaths due to cancer varying between 4.5% and 23.0% (Table 3). Cancer was also the first or second leading cause of death before age 70 years in 91 of 172 countries according to estimates from the World Health Organization (WHO) in 2015 (Bray et al., 2018). 

Based on National Cancer Registry currently, advanced cases (stage II or above) account for more than 70% of CRC, which makes curative resection as a treatment option not possible. The percentage of deaths due to CRC out of all cancer types for the years 2005 to 2018 in Brunei Darussalam varied between 9.7% and 18.4% (Table 4), higher than the global percentage of 9.2% for CRC deaths in 2018 (Bray et al., 2018). Across those thirteen years in Brunei, CRC (including cancer of the anus) was the second most common cancer-related death annually (except in years 2009 and 2010, in which this cancer type was the third most common), after cancers of the trachea, bronchus and lungs (as well as cancers of the liver and intrahepatic bile duct in years 2009 and 2010).

*Screening Practices and Rationale for National Screening*

Screening by colonoscopy or sigmoidoscopy can detect any precancerous lesions or polyps, easier in elevated polyps and more difficult in the flat or depressed variants. While the NHS BCSP issues flexible sigmoidoscopy to UK’s national screening programme, colonoscopy is the preferred screening procedure of choice in Brunei Darussalam for opportunistic CRC screening (Chong, 2014). Colonoscopy is the preferred option because sigmoidoscopy only allows the evaluation of the distal third of the colon. Therefore, any lesions (benign or neoplastic) proximal to the sigmoid and descending colon will be missed. Computed tomography colonoscopy (CTC) has been shown to be effective for CRC screening too, for cases in which the size of the lesion is larger than 10mm (Laghi et al., 2010). While CTC is an accepted modality, it is not the preferred option for screening in Brunei Darussalam due to the current heavy burden of CT scan usage for imaging of other clinical indications. CTC is generally not suitable for population screening in comparison to colonoscopy (Heitman et al., 2005). The use of radiological contrast imaging (e.g. barium enema) is obsolete in Brunei Darussalam and had been replaced by endoscopic imaging (Chong, 2014). Apart from screening CRC by direct visualisation techniques, stool-based screening is widely practiced too. Stool-based tests are designed to test for presence of minute amounts of blood in stool and are known as FOBT, of which there are two types: gFOBT and FIT, the latter being a more sensitive screening tool than the former. In addition, FIT does not require food proscription while gFOBT requires a dietary restriction beforehand as plant peroxidases, red meat and vitamin C in food interferes with gFOBT to cause false positive or negative results. In countries with screening programmes in place (either regional or national), the incidence rates of CRC with associated mortality declined by up to 30%. Furthermore, screening for CRC has been shown to be cost effective (Hassan et al., 2011; Senore et al., 2018; Sharaf and Ladabaum, 2013) in comparison to no screening, regardless of the preferred screening modalities (Patel and Kilgore, 2015). The benefits of screening will not be appreciated immediately and are seen only after more than 5-10 years of initiation of the screening programme. Over time, the proportion of CRC diagnosed in the early stages will increase. There is usually an increase in the number of cases of CRC before eventually plateauing off and even declining in the long term. 

Importantly, early detected polyps can be completely removed by effective resection to prevent progression to cancer in the typical adenoma-dysplasia-adenocarcinoma sequence. Early detection also allows the reduction in usage of adjuvant therapies that are associated with significant side effects and morbidities. According to the Brunei Darussalam National Cancer Registry, currently most CRC cases are identified at late stages, and this is associated with lowered survival rates and increased treatment cost. This is similar in Brunei Darussalam’s neighbour, Malaysia, where most CRC patients have been diagnosed at late stages and the 5-year relative survival rate by stages are lower than those in developed Asian countries (Veettil et al., 2017). CRC cases diagnosed at stages I and II have a 90% 5-year survival rate, stage III has 71% 5-year survival rate, and stage IV 14% 5-year survival rate (Bray et al., 2018). Asymptomatic cancers are usually picked up during screening that stage shifting occurs, whereby diagnosis of stage I and II cancers increases while diagnosis of stage III and IV decreases. Stage shifting is accompanied by increased survival rates and decreased morbidity, and many countries implement CRC screening programmes to ensure low morbidity and mortality rates (Schreuders et al., 2015; Worm Orntoft, 2018). 

Since 2004 in South Korea, where there is the National Cancer Screening Program (NCSP), a single FIT is provided annually for those aged ≥ 50 years, followed by a secondary confirmatory colonoscopy or double-contrast barium enema for those with positive FIT results (Yoo, 2008). Recently, the adoption of colonoscopy as a primary CRC screening tool has been argued for in South Korea, and it has been found that colonoscopy was preferred to FIT in a 2.2:1 ratio (n=396 questionnaire respondents) as the primary screening tool under NCSP (Cho et al., 2017). In Singapore, FOBT was the most cost effective screening method when compared to barium enema, colonoscopy and sigmoidoscopy (Wong et al., 2004), but if compliance to screening methods is taken into account, colonoscopy appears to be more cost effective (Vijan et al., 2001). Taiwan also launched a nationwide CRC screening programme in 2004 and the programme is consistent with the USPSTF recommendation since 2013 – the Taiwanese government provides a biennial FIT for individuals 50-75 years old (Wang et al., 2018). Similar to South Korea, those with positive FIT results are referred for follow-up examinations either with colonoscopy or double-contrast barium enema coupled with flexible sigmoidoscopy if colonoscopy is unavailable (Wang et al., 2018). Other regions in the Asia-Pacific area with a nationwide screening programme using FOBT are Australia and Japan (Koo et al., 2012). Australia factors in family history without genetic syndrome in the categorisation ofs an individual’s CRC risk and recommends different screening guidelines (including outlining the number of colonoscopies) for the average, moderate and high risk groups (Jenkins et al., 2018). The starting age for average-risk screening in Japan is set at 40 years, but like South Korea, the upper limit of screening age is not specified, and there is now awareness to obtain a colonoscopy capacity parameter to achieve an optimal screening programme (Sekiguchi et al., 2017). 

Malaysia also experiences increasing incidence of CRC but has no formal nationwide screening for CRC, relying heavily on opportunistic screening (Veettil et al., 2017). In a country with population of 32.6 million people (Malaysia Census 2019), other areas of concern to address the rising CRC burden include poor knowledge of CRC, low screening uptake, poor participation rate of primary care physicians in recommending CRC screening, and lack of optimal approaches for CRC screening (direct visualisation technique or stool-based testing or a combination of both), which would depend on availability of resources (testing tools e.g. FOBT, skilled healthcare professionals and financial support), population preferences and the use of risk-stratified scoring system (Norwati et al., 2014; Veettil et al., 2017). In a 2006 survey, poor knowledge and poor physician recommendation rate were also observed in Singapore, which is the only member state of ASEAN that is considered developed (Koo et al., 2012). 

The largest known, one-time off CRC screening programme in Brunei Darussalam was conducted in 2009 (Chong et al., 2013). This CRC screening was part of the National Integrated Health Screening for government servants that ran from 2009 to 2013. CRC screening was done through a single FIT (n=11,576, of which 7,360 [66.9%] returned the specimen) and an assessment of family or personal history of CRC or neoplasms. Those positive for either or both (FIT positive, n=142 [1.9%]; positive history for CRC, n=329 [2.8%]; positive history for polyps, n=135, [1.2%]) were referred for further evaluation. Only 17.7% of those referred (n=107) attended screening assessment, and of this only 50.4% (n=54) agreed for colonoscopy. CRC were detected in 13.0% (n=7), and all advanced lesions were detected in patients with positive FIT. While uptake of screening colonoscopy was low at 50.4%, the programme showed that screening for CRC with a single FIT was effective. 

Nevertheless, the success of a screening programme is largely dependent upon the uptake of screening. In a multicentre, comparative study involving 14 regions/countries from the Asia-Pacific region (Australia, Brunei Darussalam, China, Hong Kong, India, Indonesia, Japan, Korea, Malaysia, Pakistan, Philippines, Singapore, Taiwan and Thailand), physician recommendation to patients for screening adherence as well as knowledge of CRC and screening tests were found to be the most important predictors of screening participation across all 14 areas (Koo et al., 2012). The Netherlands began a national population screening in 2014 and showed the highest participation rate (68.2%) while some areas of Canada showed the lowest participation rate (16.0%) from 2009 to 2011 (Navarro et al., 2017). Japan is an early pioneer with its CRC screening programme in place since 1992 (Navarro et al., 2017), but national average participation rates in Japan were 24.8% in 2010 (Hashimoto et al., 2014) and 21.1% in 2012 (Hamashima and Sano, 2018). Therefore, current uptake in any existing screening programmes have not been optimal and can be further improved (Benton et al., 2017; Hamashima and Sano, 2018; Kerrison et al., 2018). Even so, benefits of such programmes are visible. Hence, there ought to be more strategies in place to advocate CRC prevention through the promotion of compliance for screening uptake. For example, intensive one-to-one education of patient on FOBT increases the chances of a patient returning specimens compared to small media, for example, the handing out of educational materials that are written down (Brouwers et al., 2011; Stokamer et al., 2005). The time spent by healthcare providers making explanations on the need for FIT and on how to collect stool specimens would increase compliance to stool-based CRC screening in the community. Across low-income communities in Malaysia, it was possible to achieve a return rate of FIT specimens of 91.2% and a compliance rate for colonoscopy of 70.3% from 2010 to 2015 despite lack of knowledge about CRC and stool testing among participants (Ng et al., 2016). The high rates could be attributed to multiple rounds of door-to-door knocks as well as close follow-up with residents regarding FIT results and further medical management with colonoscopy, so that the participants would be able to follow through initial to subsequent screening steps (Ng et al., 2017). Other strategies of interest to consider include electronic text messaging of scheduled appointments to patients (Camilloni et al., 2013; Sequist et al., 2011) from the Brunei Darussalam Healthcare Information and Management Systems (Bru-HIMS).

When considering a national CRC screening programme, three factors must be taken into account. Firstly, the incidence of CRC must be both sufficiently high and increasing for screening to be cost effective. The incidence of CRC in Brunei Darussalam has increased (Chong et al., 2009; Chong et al., 2015b; Ong et al., 2018). Furthermore, most are still diagnosed at the advanced stages but CRC can be prevented by earlier detection and removal of polyps. Secondly, the technical and financial support must be considered. The Ministry of Health in Brunei Darussalam is committed to addressing the scourge of CRC and other non-communicable diseases, and is currently in the process of implementing a national screening programme for metabolic diseases collectively (including cardiac diseases, diabetes mellitus, dyslipidaemia, hypertension, dyslipidaemia and obesity). Thirdly, public participation is important. Our own experience showed that public awareness and knowledge on CRC remains poor (Chong et al., 2015a). Therefore, public education to raise awareness is important and will be incorporated in the proposed screening programme.

The Ministry of Health in Brunei Darussalam is currently in the process of implementing a CRC screening programme as part of a larger national health screening based on the increasing incidence of non-communicable diseases. A review to report the progress and results of the national screening programme at the population level will be available after few years, at which point the benefits of screening will come to fruition. With the implementation of a CRC screening programme, it is expected that the incidence of CRC will plateau, and that both the proportion of advanced diseases and CRC associated mortality will decline. Like any screening programmes, there will be issues encountered and teething problems, such as low levels of participations. Nonetheless, with time, these issues will be resolved alongside improvements to the screening programme.

In conclusion, with the increasing incidence of CRC worldwide and in Brunei Darussalam, screening for CRC is now an important issue. While formal screening programmes either regionally or nationally are not available in many nations, screening has been shown to reduce the incidence of CRC related mortality and to eventually result in the decline of CRC incidence. Opportunistic screening is widely practiced but this is not standardised nor coordinated. The Ministry in Health of Brunei Darussalam is collaborating with other government and non-government agencies to embark with the setup of a national screening programme which incorporates CRC screening. The proposed programme will utilise colonoscopy for those with family history of CRC, as well as FIT screening and subsequent colonoscopy for those FIT-positive. A review of the progress and outcome of the national screening programme will be available a few years after rollout.
